# *Proalesamplus* sp. nov., a new monogonont rotifer with a large epipharynx from Korea (Rotifera, Proalidae)

**DOI:** 10.3897/BDJ.12.e129622

**Published:** 2024-10-14

**Authors:** Hee-Min Yang, Gi-Sik Min

**Affiliations:** 1 Department of Biological Sciences and Bioengineering, Inha University, Incheon 22212, Republic of Korea Department of Biological Sciences and Bioengineering, Inha University Incheon 22212 Republic of Korea; 2 Research Institute of EcoScience, Ewha Womans University, Seoul 03760, Republic of Korea Research Institute of EcoScience, Ewha Womans University Seoul 03760 Republic of Korea; 3 National Institute of Biological Resources, Incheon 22689, Republic of Korea National Institute of Biological Resources Incheon 22689 Republic of Korea

**Keywords:** malleate, Monogononta, new species, SEM, taxonomy

## Abstract

**Background:**

The family Proalidae Harring & Myers, 1924, includes four genera and 53 species, distributed across all eight biogeographic realms and inhabiting various environments, including freshwater, saltwater and terrestrial environments. The genus *Proales*, the largest within Proalidae, encompasses 41 species characterised by diverse morphological traits. In Korea, the presence of Proalidae has been documented with five known species: *Bryceellaperpusilla* Wilts, Martínez Arbizu & Ahlrichs, 2010, *B.stylata* (Milne, 1886), *B.tenella* (Bryce, 1897), *Proalesfallaciosa* Wulfert, 1937 and *Proalinopsiscaudatus* (Collins, 1872).

**New information:**

A new species, *Proalesamplus* sp. nov., is the 42^nd^ species within the genus *Proales*. This species exhibits unique morphological characteristics in the trophi, particularly in the epipharynx, which are distinctive enough to prevent misidentification with other *Proales* species. The habitus of the new species bears some resemblance to *P.phaeopis* Myers, 1933, sharing features, such as an elongated and fusiform body, two eyespots, a single foot pseudosegment, two short toes and the absence of a dorsal papilla between the toes. However, the epipharynx of the two species is markedly different. The unique epipharynx characteristic of this new species is unparalleled within the genus *Proales*.

## Introduction

The family Proalidae Harring & Myers, 1924 comprises four genera and 53 species: *Bryceella* Remane, 1929; *Proales* Gosse, 1886; *Proalinopsis* Weber, 1918; and *Wulfertia* Donner, 1943 (Jersabek and Leitner 2013). These species exhibit a cosmopolitan distribution and inhabit a wide range of habitats from freshwater to saltwater, including some terrestrial environments. Most proalid species are free-living, but certain species show parasitic tendencies towards organisms, such as hydra, crab, algae or colonial ciliates (Remane 1929, Thane-Fenchel 1966, May 1989, De Smet 1996, Wallace et al. 2001).

This taxon was first erected as the subfamily Proalinae within the family Notommatidae Hudson & Gosse, 1886, characterised by its malleate type trophi (Harring and Myers 1924). It was later elevated to family level by Bartoš (1959). Within the phylum Rotifera Cuvier, 1817, the family Epiphanidae Harring, 1913 is also characterised by an illoricated or semi-located body and malleate-type trophi. However, these two families are distinguished by the morphological characteristics of the corona, mouth and trophi (Wallace et al. 2006). In the Proalidae, the corona is situated obliquely, with the mouth typically located at or near the ventral margin of the buccal field. The trophi in Proalidae can be of three types: malleate, modified malleate and virgate. In contrast, in Epiphanidae, the corona is situated apically, with the mouth located at the funnel of the buccal field and the trophi is exclusively of the malleate type (Segers 2004).

Within Proalidae, the genus *Proales* is the largest genus, comprising 41 species (Jersabek and Leitner 2013). Representatives of this taxon are morphologically characterised by its illoricated or rarely semi-loricated body, along with trophi of the malleate, modified malleate and virgate-types. Nevertheless, while other genera within Proalidae exhibit unique characteristics, such as the corona with long cirri in *Bryceella*, a papilla bearing a tuft of setae in *Proalinopsis* and a notably reduced corona in *Wulfetia*, *Proales* lacks genus-specific characteristics (De Smet 1996). For this reason, *Proales* is considered a group of species with diverse characteristics, underscoring the taxonomic challenges within the genus and emphasising the necessity for ongoing re-assessment and classification. Recently, several species within *Proales* have been reclassified to other genera, based on morphological and molecular data: *Proaleswerneckii* (Ehrenberg, 1834) was re-assigned to the genus *Pourriotia* De Smet, 2003, based on the morphological characteristics of its trophi; *Proalessigmoidea* (Skorikov, 1896) to the genus *Pleurotrocha* Ehrenberg, 1830, based on morphological and ecological characteristics; and *Proalesdaphnicola* Thompson, 1892 to the genus *Epiphanes* Ehrenberg, 1832, based on morphological characteristics and molecular analysis (De Smet 2009, Wilts et al. 2009, Wilts et al. 2012).

In Korea, the presence of the family Proalidae was first documented by Song and Jin (2000) with the report of *Bryceellatenella* (Bryce, 1897). Subsequent research has increased the total number of the proalid species to five, including *B.perpusilla* Wilts, Martínez Arbizu & Ahlrichs, 2010, *B.stylata* (Milne, 1886), *Proalesfallaciosa* Wulfert, 1937 and *Proalinopsiscaudatus* (Collins, 1872) (Song 2015, Song 2017, Yang and Min 2022). This study contributes to the knowledge by identifying a new *Proales* species collected from a soil sample in Korea, detailing morphological characteristics and partial gene sequences of mitochondrial cytochrome *c* oxidase subunit I (COI), nuclear 18S rDNA, 28S rDNA and internal transcribed spacer 1 (ITS1).

## Materials and methods

### Sampling and morphological observation

**Figure 1.**
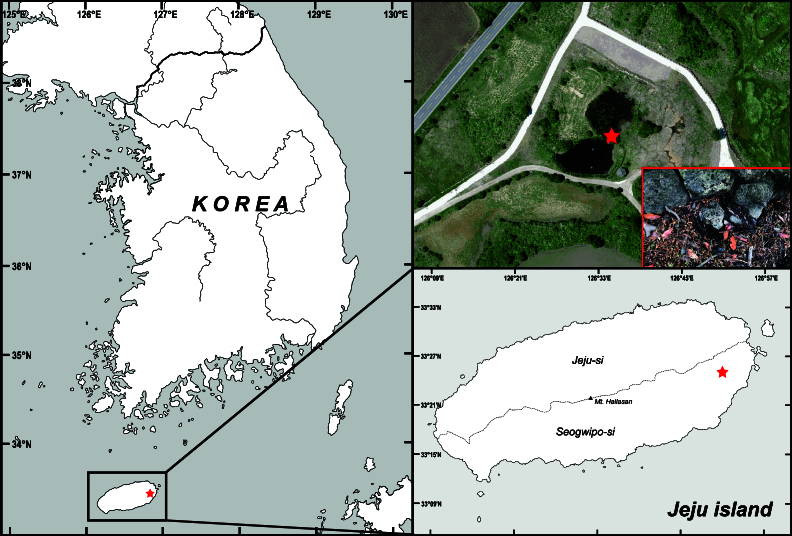
Map and habitat showing the collection sites of *Proalesamplus* sp. nov. in this study.

Rotifer specimens examined in this study were isolated from a soil sample collected from the edge of a pond on Jeju Island, Korea (Fig. 1). The soil samples were air-dried for several weeks, after which a portion of them was transferred to a new plant culture dish (310100, SPL Life Science, Korea) and rewetted using mineral water. At room temperature, rotifers were discovered approximately one week after rewetting. The rotifers were transferred to a new culture dish under a stereomicroscope (SZX7, Olympus, Japan). For live specimen observations, a few drops of 1% bupivacaine solution (B5274, Sigma-Aldrich, USA) were used to ananesthetise the rotifers. Photographs and videos of live specimens were taken using a digital camera equipped with an optical microscope (DM2500, Leica, Germany). Permanent microscope slides were prepared by fixing the anaesthetised rotifers in 4% formaldehyde and mounting them in glycerol.

For trophi observation, the trophi were isolated using a commercial bleach containing 4–5% sodium hypochlorite (Yuhan-Chlorox, Korea) and prepared for scanning electron microscopy (SEM) according to the methods of De Smet (1998). Whole body specimens for SEM were prepared as follows: (1) the rotifers were fixed in a 2% osmium tetroxide (OsO_4_) solution (O5500, Sigma-Aldrich, USA) for 30 minutes and then rinsed with distilled water; (2) the rotifers were dehydrated in an ascending series of ethanol concentrations (30%, 50%, 70%, 90%, 95%, 100%, 10 minutes for each step); (3) 100% ethanol was replaced with hexamethyldisilazane (440191, Sigma-Aldrich, USA) twice, with 10 minutes for each step; (4) the rotifers were transferred to a coverslip and allowed to dry overnight (Shively and Miller 2009). The SEM instrument used for observation was the SU8010 (Hitachi, Japan), with an accelerating voltage of 5 kV for whole-body observations and 10 kV for trophi observations. The ImageJ 1.53k software (Abràmoff et al. 2004) was used to measure the body and trophi elements. All examined specimens were deposited in the specimen repository of the National Institute of Biological Resources (NIBR), Korea.

### DNA sequencing and molecular analysis

Using the LaboPass^TM^ Tissue Genomic DNA Isolation Kit Mini (Cosmo Genetech, Korea), three genomic DNAs were extracted, each from three different individuals. PCR was conducted using TaKaRa Ex Taq^®^ (TaKaRa, Japan) in a final volume of 25 µl under the following conditions: an initial denaturation at 94 °C for 5 minutes, followed by 40 cycles of denaturation at 94 °C for 30 seconds, annealing at 51 °C for 30 seconds, extensions at 72 °C for 1 minute and a final extension at 72 °C for 5 minutes. For PCR targeting 18S rDNA, the extension time was extended to 2 minutes. The primer sets used for the PCR are listed in Table 1. The PCR products were purified using a LaboPass^TM^ PCR Purification Kit (Cosmo Genetech) and subsequently sequenced at Macrogen (Korea). The obtained sequences were trimmed and aligned using Geneious ver. 8.1.9 (https://www.geneious.com). The genetic distances were calculated using MEGA ver. 11 (Tamura et al. 2021) with *p*-distance methods. The Maximum-Likelihood (ML) phylogenetic tree, based on 18S rDNA sequences, was inferred using IQ-TREE ver. 2.3.1 (Minh et al. 2020), with the GTR+F+I+R2 model selected according to the Akaike Information Criterion (AIC) by ModelFinder (Kalyaanamoorthy et al. 2017). For the SH-like approximate likelihood ratio test (Guindon et al. 2010) and ultrafast bootstrap (Hoang et al. 2018), 1,000 bootstrap replicates were performed. The ML tree was visualised using FigTree ver. 1.4.4 (http://tree.bio.ed.ac.uk) and modified using Illustrator 2021 (Adobe, USA). The sequences used for molecular analysis are listed in Table 2.

## Taxon treatments

### Proales amplus

Yang & Min sp. nov.

A4770A82-56BE-513D-9D55-46E6A6F57B40

9EF7345C-3E92-4DA3-81FF-B3899F1EE635

#### Materials

- **Type status:** Holotype. **Occurrence:** catalogNumber: NIBRIV0000909850; occurrenceID: CBCF2B2C-0172-52CB-A556-4677FBD3267E; **Taxon:** scientificName: *Proalesamplus*; phylum: Rotifera; family: Proalidae; genus: Proales; scientificNameAuthorship: Yang & Min; **Location:** island: Jeju island; country: South Korea; verbatimLatitude: 33°25'13"N; verbatimLongitude: 126°50'42"E; **Event:** eventDate: 09 Jan. 2022; **Record Level:** institutionCode: National Institute of Biological Resources (NIBR)
- **Type status:** Paratype. **Occurrence:** catalogNumber: NIBRIV0000909851; occurrenceID: 62604C3B-E7C0-5DBE-BB2B-211A1E1D63A9; **Taxon:** scientificName: *Proalesamplus*; phylum: Rotifera; family: Proalidae; genus: Proales; scientificNameAuthorship: Yang & Min; **Location:** island: Jeju island; country: South Korea; verbatimLatitude: 33°25'13"N; verbatimLongitude: 126°50'42"E; **Event:** eventDate: 09 Jan. 2022; **Record Level:** institutionCode: National Institute of Biological Resources (NIBR)
- **Type status:** Paratype. **Occurrence:** catalogNumber: NIBRIV0000909852; occurrenceID: 16040B1E-09DA-51CB-8EC7-8664FE3064AB; **Taxon:** scientificName: *Proalesamplus*; phylum: Rotifera; family: Proalidae; genus: Proales; scientificNameAuthorship: Yang & Min; **Location:** island: Jeju island; country: South Korea; verbatimLatitude: 33°25'13"N; verbatimLongitude: 126°50'42"E; **Event:** eventDate: 09 Jan. 2022; **Record Level:** institutionCode: National Institute of Biological Resources (NIBR)
- **Type status:** Paratype. **Occurrence:** catalogNumber: NIBRIV0000909853; occurrenceID: 757532B6-2E54-56B4-BD14-011208DFCD69; **Taxon:** scientificName: *Proalesamplus*; phylum: Rotifera; family: Proalidae; genus: Proales; scientificNameAuthorship: Yang & Min; **Location:** island: Jeju island; country: South Korea; verbatimLatitude: 33°25'13"N; verbatimLongitude: 126°50'42"E; **Event:** eventDate: 09 Jan. 2022; **Record Level:** type: SEM preparation; institutionCode: National Institute of Biological Resources (NIBR)
- **Type status:** Paratype. **Occurrence:** catalogNumber: NIBRIV0000909854; occurrenceID: 75CFAB69-4632-5DE3-A047-B4CE6C810DC3; **Taxon:** scientificName: *Proalesamplus*; phylum: Rotifera; family: Proalidae; genus: Proales; scientificNameAuthorship: Yang & Min; **Location:** island: Jeju island; country: South Korea; verbatimLatitude: 33°25'13"N; verbatimLongitude: 126°50'42"E; **Event:** eventDate: 09 Jan. 2022; **Record Level:** type: SEM preparation; institutionCode: National Institute of Biological Resources (NIBR)

#### Description

**Figure 2.**
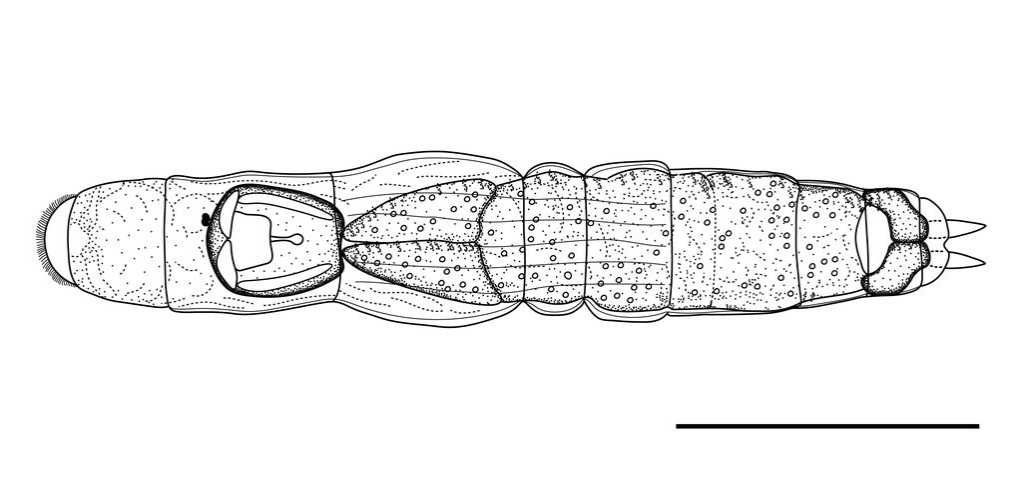
Line drawing of *Proalesamplus* sp. nov., dorsal view. Scale bar: 100 μm.

**Figure 3.**
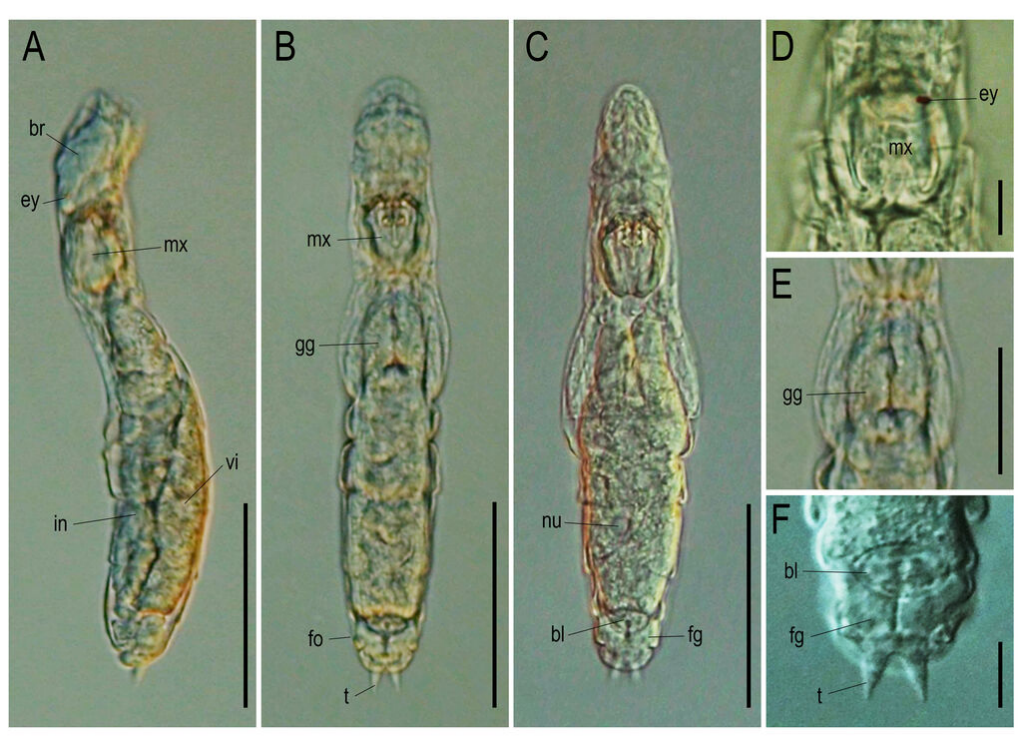
Live specimen of *Proalesamplus* sp. nov. observed under the optical microscope. **A** lateral view; **B** dorsal view; **C** ventral view; **D** eyespots and mastax; **E** gastric glands, dorsal view; **F** bladder and foot glands. Scale bars: A–C = 100 μm, D, F = 20 μm, E = 50 μm. Abbreviations: bl = bladder, br = brain, ey = eyespots, fg = footglands, fo = foot, gg = gastric glands, in = intestine, mx = mastax, nu = nuclei, t = toe, vi = vitellarium.

**Figure 4.**
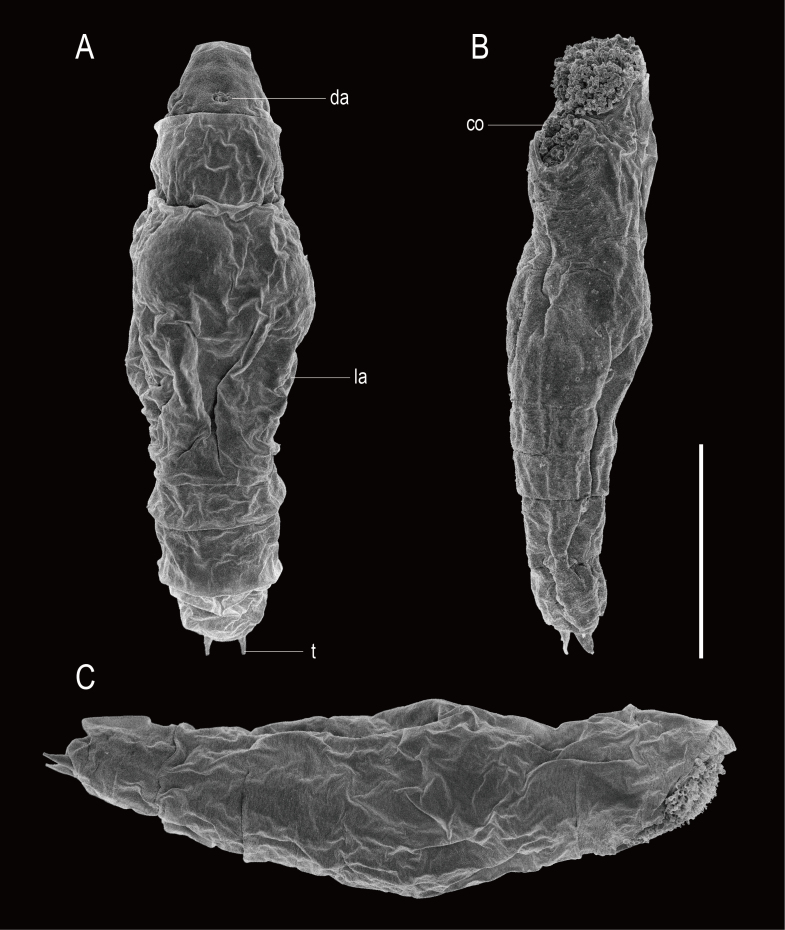
SEM image of the habitus of *Proalesamplus* sp. nov. **A** dorsal view; **B** ventral view; **C** lateral view, right side. Scale bar: 30 μm. Abbreviations: co = corona, da = dorsal antenna, la = lateral antenna, t = toe.

**Adult Female.** Total length 277–303 μm (n = 5). Illoricated body soft, flexible, hyaline. Body slender, cylindrical, fusiform in dorsal and lateral views (Figs 2, 3, 4). Head distinguished from trunk by transverse fold in dorsal view. One dorsal antenna located near posterior fourth of head (Fig. 4A). Rostrum short, broad, semicircle (Fig. 4C). Corona oblique or slightly ventral, extending from anterior margin to ventral side of head (Fig. 4B and C). Brain saccate, large, occupying most of the head region dorsally (Figs 2, 3A). Retrocerebral sac absent. Two tiny red eyespots on the brain, positioned very close together and displaced to the right (Fig. 3D). Trunk with six transverse folds, several longitudinal folds dorsally in the middle. A pair of lateral antennae at the middle of trunk (Fig. 4A). Tail short, wide, distally rounded, covering half of foot. Foot with one pseudosegment, short, approximately 1/20 of total length. Toes symmetrical, short, slim, blunt ends, 10–11 μm in length (Fig. 3F). Toes straight in dorsal view, slightly curved upwards in lateral view (Fig. 3C). Pedal glands symmetrical, large, kidney-shaped, exceeding beyond foot length. Vitellarium with eight nuclei.

**Digestive organs.** Mouth somewhere at the posterior end of the ventral side of the corona. Oesophagus thin, long, passing between brain and mastax, connecting mouth and stomach along dorsal side (Suppl. material 1). Both stomach and intestine hyaline, difficult to distinguish. Gastric glands very large, elongated pyriform, located antero-dorsal to stomach (Fig. 3E). Bladder oval-shaped, moderate size when filled. Salivary glands and cloaca not observed.

**Figure 5.**
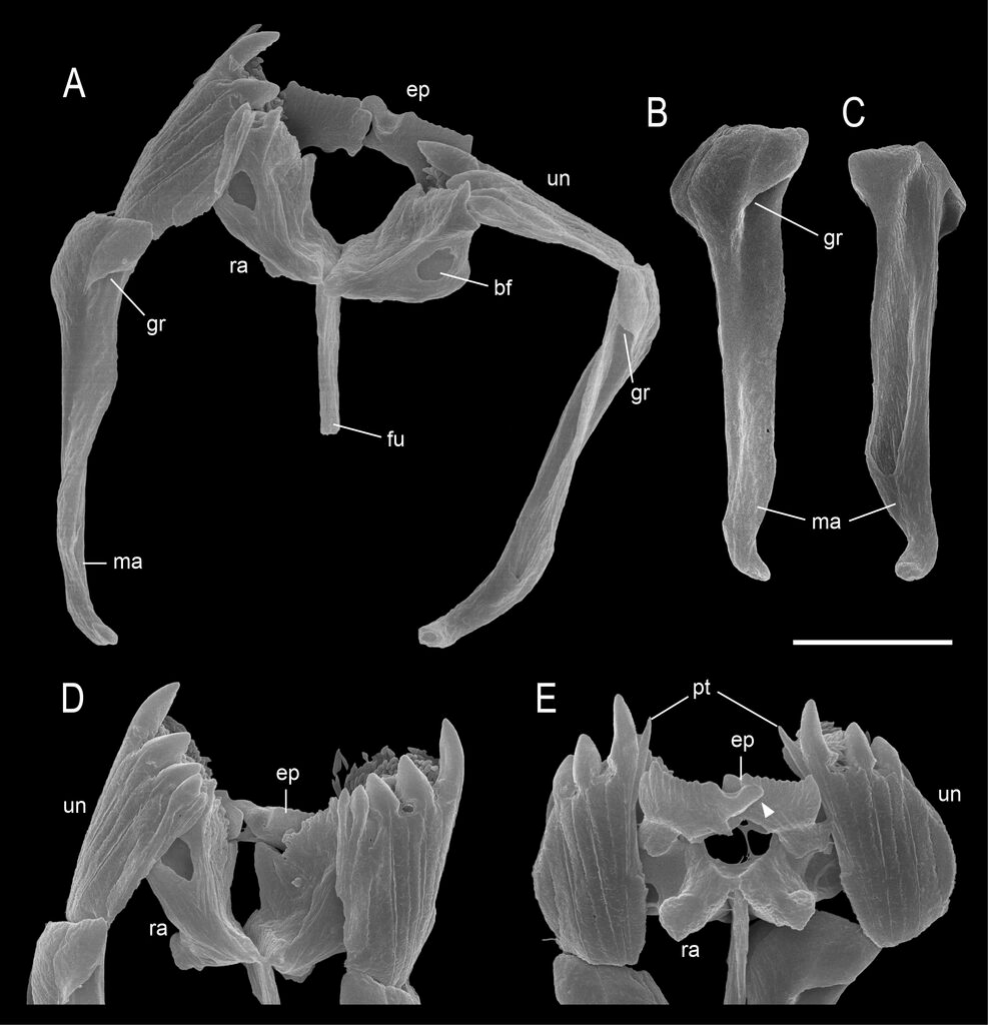
SEM image of the trophi of *Proalesamplus* sp. nov. **A** dorsal view; **B** left manubrium, outside view; **C** left manubrium, inside view; **D** unci and rami, dorsal view; **E** unci and rami, ventral view, arrow indicates the extension of the right epipharynx. Scale bar: 5 μm. Abbreviations: bf = basifenestra, ep = epipharynx, fu = fulcrum, gr = groove, ma = manubrium, pt = pre-uncinal tooth, ra = ramus, un = uncus.

**Trophi.** Malleate type, almost symmetrical (Fig. 5A). Rami without alulae on lateral side in dorsal view; ventral view with a pair of blunt projections proximally; inner margin smooth, without projections or scleropili; a pair of oval-shaped basifenestrae in the middle on both dorsal sides of ramus (Fig. 5A and E). Fulcrum short, thin, straight, rod-shaped in dorso-ventral view, without expansion at posterior end; 5.0–6.1 μm in length, approximately 1/3 the length of manubria (Fig. 5A) Unci 7.5–8.4 μm in length; symmetrical with five teeth; teeth composed of three large and two small, increasing in size dorsally to ventrally; pre-uncinal tooth at the largest unci teeth (Fig. 5D and E). Manubria 13.1–14.9 μm in length; symmetrical, clubbed shape, gradually narrowing anteriorly to posteriorly; anterior end blunt, slightly expanded; one groove located anteriorly on dorsal side; middle part of manubria slightly twisted outwards; posterior end blunt, sharply tapered, curved dorsally (Fig. 5B and C). Epipharynx asymmetrical, large, wide and flat; anterior margin comb-like-shaped; posterior base with a width of 1/3 of anterior margin, extending to the middle of the ventral side of rami; proximal anterior side of right epipharynx extending towards left (Fig. 5A and E).

Male and eggs unknown.

#### Diagnosis

Body slender, fusiform. Head distinguished from trunk by transverse fold. Two tiny red eyespots on the brain positioned very close together displaced to right. Trunk with six transverse folds, several longitudinal folds dorsally in the middle. Foot short, with one pseudosegment. Toes short, slim, ending in blunt tips. Pedal glands large, exceeding foot in length. Gastric glands very large, elongated, pyriform. Trophi malleate. Rami without alulae, with a pair of blunt projections located proximally. Fulcrum short, thin, rod-shaped in dorso-ventral view. Unci with five teeth, comprising three large teeth and two small teeth. Preuncinal tooth at the largest unci teeth. Manubria club-shaped, slightly twisted, posterior end curved dorsally. Epipharynx large, wide, flat, with comb-like shape at apical margin; posterior base with a width of 1/3 of anterior margin, extending to the middle of the ventral side of rami; proximal anterior side of right epipharynx extending towards left.

#### Etymology

The specific name amplus is derived from the Latin word *amplus*, meaning large or broad, referring to the size and shape of the epipharynx.

#### Molecular data

Partial sequences of four genes (COI, 18S rDNA, 28S rDNA and ITS1) from three specimens were obtained. Intraspecific variations were not found in any of the genes. The corresponding GenBank accession numbers for each of these gene sequences are as follows: COI, 660 bp (PP750787–PP750789); 18S rDNA, 1644 bp (PP751790–PP751792); 28S rDNA, 780 bp (PP751742–PP751744); and ITS1, 324 bp (PP751753–PP751755).

#### Phylogenetic analysis

To calculate the genetic distances of *Proales* species, five sequences each of COI and 18S rDNA, and four sequences of 28S rDNA were used (Table 2). The genetic distances between the *Proales* species ranged from 0.249–0.340 for COI, 0.018–0.041 for 18S rDNA and 0.160–0.254 for 28S rDNA (Table 3). The new species exhibited significant genetic distances from other *Proales* species in all three genes, supporting that the species is not only morphologically, but also molecularly a new species. As for ITS1, it was not possible to calculate the genetic distances between *Proales* species, as this marks the first online presence of ITS1 sequences for Proalidae. Given the recent use of the ITS1 region as a useful marker in species diversity studies, we have provided ITS1 sequences for the new species (Papakostas et al. 2016, Kordbacheh et al. 2017, Mills et al. 2017, Kordbacheh et al. 2018).

**Figure 6.**
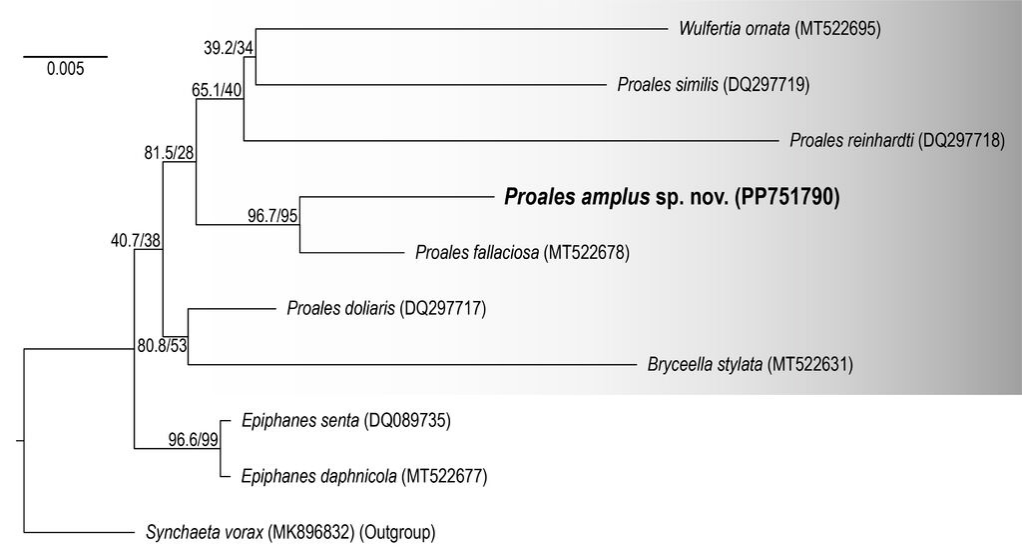
Maximum-Likelihood (ML) tree of Proalidae and *Epiphanes* species, based on 18S rDNA, using the GTR+F+I+R2 model. Values on the nodes represent SH-like approximate likelihood ratio and ultrafast bootstrap support. The scale bar indicates branch length as the number of nucleotides substitutions per site.

The 18S rDNA Maximum-Likelihood (ML) phylogenetic tree was constructed, based on seven Proalidae species, two *Epiphanes* species and one *Synchaeta* Ehrenberg, 1832 as an outgroup (Fig. 6, Table 2). The new species, *Proalesamplus* sp. nov., formed a clade with *P.fallaciosa* (MT522678) with high support (SH-aLRT = 96.7, bootstrap value = 95). Within the Proalidae clade, the genus *Proales* did not form a monophyletic group. *Bryceellastylata* (MT522631) formed a clade with *P.doliaris* (DQ297717) and *Wulfertiaornata* (MT522695) formed a clade with *P.similis* (DQ297719), both with low support values. *Epiphanesdaphnicola*, formerly classified as *Proales*, was clearly separated from the other Proalidae species and formed a clade with *Epiphanessenta* (DQ089735) with high support (SH-aLRT = 96.6, bootstrap value = 99), consistent with the previous results of Wilts et al. (2012).

## Discussion

The new species described in this study, *Proalesamplus* sp. nov., is the 42^nd^ species within the genus *Proales*. This species exhibits unique morphological characteristics in the trophi, particularly in the epipharynx, which are distinctive enough to prevent misidentification with other *Proales* species (Fig. 5). The habitus of the new species bears some resemblance to *P.phaeopis* Myers, 1933, sharing features such as an elongated and fusiform body, a single foot pseudosegment, two short toes, two eyespots and the absence of a dorsal papilla between the toes. However, the epipharynx of the two species is markedly different (Myers 1933). To our knowledge, the unique epipharynx characteristic of this new species is unparalleled within the genus *Proales*. While *Proales* is known for its broad diversity in epipharynx shapes and sizes (De Smet 1996), this new species distinguishes itself with a large, flat epipharynx featuring a comb-like shape on the apical margin, deviating notably from the typical epipharynx variations observed within the genus.

Within the family Proalidae, this type of large and flat epipharynx is not exclusive to the new species; similar structures are observed within the genus *Wulfertia*. Each of the three species in *Wulfertia* is characterised by large epipharynx plates with serrated or bluntly serrated apical margins (Donner 1943, Koste and Tobias 1990, De Smet and Bafort 1992). Despite these morphological similarities, particularly in epipharynx shape, the new species is not classified within *Wulfertia* due to its distinct corona morphology. Unlike the markedly reduced corona observed in *Wulfertia*, the new species displays an oblique or slightly ventral corona without such reduction. This distinction in corona morphology is a pivotal factor in its classification outside of *Wulfertia* (Donner 1943).

In the molecular analysis (Fig. 6, Table 3), the new species showed a significant genetic distance from other species, enough to be considered a distinct species from them. Additionally, in the phylogenetic tree, it formed a clade with other species in the Proalidae, supporting its genetic allocation within the genus *Proales*. However, *Proales* did not form a monophyletic group and exhibited a long branch tendency. We believe this is due to the lack of genetic information within Proalidae. Within the family Proalidae, 53 species have been recorded worldwide. However, molecular data are available for only eight species: one species of *Bryceella*, five species of *Proales* and one species of *Wulfertia*.

The limited availability of molecular data, particularly considering the taxonomic complexity of the genus *Proales*, highlights the critical need for more comprehensive taxonomic research. The genus *Proales* is recognised as a complex group, making molecular data particularly important in resolving its intricate taxonomic challenges. In the study proposing the re-assignment of *E.daphnicola* to the genus *Epiphanes*, molecular data played a crucial role as key evidence (Fig. 6, Wilts et al. (2012)). The continuous acquisition of molecular data from a diverse range of species within the Proalidae will not only elucidate phylogenetic relationships, but also facilitate the identification and classification of species and support the discovery and reclassification of species.

## Supplementary Material

XML Treatment for Proales amplus

9ACFEAD3-32D7-5B7F-B472-DE6C7F97F4C9 10.3897/BDJ.12.e129622.suppl1 Supplementary material 1 Proalesamplus sp. nov. lateral view of head Data type video File: oo_1115683.mp4 https://binary.pensoft.net/file/1115683 Hee-Min Yang and Gi-Sik Min

## Figures and Tables

**Table 1.** PCR primer sets utilised in this study.

**Gene**	**Primer**	**Sequence (5’-3’)**	**Reference**
COI	LCO1490	GGTCAACAAATCATAAAGATATTGG	Folmer et al. (1994)
	HCO2198	TAAACTTCAGGGTGACCAAAAAAT	
18S	EukA	AACCTGGTTGATCCTGCCAGT	Medlin et al. (1988)
	EukB	TGATCCTTCTGCAGGTTCACCTAC	
28S	D1F	GGGACTACCCCCTGAATTTAAGCAT	Crandall et al. (2000) Park and O'Foighil (2000)
	Rd4b	CCTTGGTCCGTGTTTCAAGAC	
ITS1	III	CACACCGCCCGTCGCTACTACCGATTG	Palumbi (2006)
	VIII	GTGCGTTCGAAGTGTCGATGATCAA	

**Table 2.** List of species and corresponding GenBank accession numbers for molecular analyses.

**Gene**	**Species**	**GenBank No.**	**Reference**
COI	*Proalesamplus* sp. nov.	PP750787	This study
	*Proalesdoliaris* (Rousselet, 1895)	DQ297790	Sørensen and Giribet (2006)
	*Proalesfallaciosa*	HQ873041	Wilts et al. (2012)
	*Proalessimilis* De Beauchamp, 1907	DQ297791	Sørensen and Giribet (2006)
	*Proalestheodora* (Gosse, 1887)	HQ873043	Wilts et al. (2012)
18S	*Proalesamplus* sp. nov.	PP751790	This study
	*Proalesdoliaris*	DQ297717	Sørensen and Giribet (2006)
	*Proalesfallaciosa*	MT522678	Bininda-Emonds (2021)
	*Proalesreinhardti* (Ehrenberg, 1834)	DQ297718	Sørensen and Giribet (2006)
	*Proalessimilis*	DQ297719	Sørensen and Giribet (2006)
	*Bryceellastylata*	MT522631	Bininda-Emonds (2021)
	*Wulfertiaornata* Donner, 1943	MT522695	Bininda-Emonds (2021)
	*Epiphanesdaphnicola* (Thompson, 1892)	MT522677	Bininda-Emonds (2021)
	*Epiphanessenta* (Müller, 1773)	DQ089735	García-Varela and Nadler (2006)
	*Synchaetavorax* Rousselet, 1902	MK896832	Wilke et al. (2020)
28S	*Proalesamplus* sp. nov.	PP751742	This study
	*Proalesdoliaris*	DQ297753	Sørensen and Giribet (2006)
	*Proalesreinhardti*	DQ297754	Sørensen and Giribet (2006)
	*Proalessimilis*	DQ297755	Sørensen and Giribet (2006)

**Table 3.** Genetic distances of the genus *Proales* calculated via the *p*-distance method using three molecular markers (COI, 18S rDNA, 28S rDNA).

**COI**								
	**Species**	**GenBank No.**	**1**	**2**		**3**		**4**
1	*Proalesamplus* sp. nov.	PP750787						
2	*Proalesdoliaris*	DQ297790	0.296					
3	*Proalesfallaciosa*	HQ873041	0.264	0.296				
4	*Proalessimilis*	DQ297791	0.249	0.340		0.250		
5	*Proalestheodora*	HQ873043	0.283	0.328		0.252		0.293
**18S rDNA**								
	**Species**	**GenBank No.**	**1**	**2**		**3**		**4**
1	*Proalesamplus* sp. nov.	PP751790						
2	*Proalesdoliaris*	DQ297717	0.022					
3	*Proalesfallaciosa*	MT522678	0.018	0.019				
4	*Proalesreinhardti*	DQ297718	0.040	0.032		0.033		
5	*Proalessimilis*	DQ297719	0.031	0.025		0.030		0.041
**28S rDNA**								
		**GenBank No.**	**1**		**2**		**3**	
1	*Proalesamplus* sp. nov.	PP751742						
2	*Proalesdoliaris*	DQ297753	0.167					
3	*Proalesreinhardti*	DQ297754	0.188		0.160			
4	*Proalessimilis*	DQ297755	0.247		0.229		0.254	
